# Do Italian people still wear masks? Analysis of personality and dispositional correlates of facemask use in post Covid-19 scenario

**DOI:** 10.1038/s41598-023-43588-8

**Published:** 2023-10-02

**Authors:** Paola Rigo, Marina Miscioscia, Silvia Spaggiari, Daniela Di Riso

**Affiliations:** Department of Developmental and Socialization Psychology (DPSS), University of Padua, 35131 Padova, Italy

**Keywords:** Psychology, Human behaviour

## Abstract

Face mask wearing is a low-cost preventative measure for the Covid-19 pandemic. In Italy, face masks are no longer mandatory indoors from the 1^st^ of May 2022. Some research focused on factors that influence the choice of using masks, but less is known about mask-wearing when non-mandatory. The present study aims to compare those who were still wearing masks indoors when non-mandatory and those who were not, in personality traits, anxiety, depression, and trust in healthcare professions, in Italy, in 2022. Furthermore, we analyze if resilience, reactance, political orientation, and Covid-19 vaccinations moderate between negative affectivity and the choice of wearing masks. 1151 adults, aged 18–64, were recruited. Using the Qualtrics platform, participants filled in a socio-demographic interview, and self-report questionnaires. Results showed that people who were still wearing a mask indoors had higher levels of psychoticism and negative affectivity, worse mental health, greater trust in healthcare professions, and worries about the pandemic. Moreover, resilience partially moderates the relationship between negative affectivity and the choice of wearing a mask. These findings provide a better understanding of individuals’ responses to post-pandemic changes, identifying the personal and contextual aspects that can make people struggle with the process of returning to normality.

## Introduction

The Covid-19 pandemic has had a severe global impact. Institutions and governments struggled to find ways to limit the spread of the virus. One of the main and low-cost preventative measures is wearing face masks, as personal protective equipment (PPE), to prevent droplets from spreading in the air^1^. Although most of the evidence showed that wearing face masks is an effective way to reduce virus transmission, a debate was triggered, and many people expressed resistant approaches to the mandated use^2^. To our knowledge, what has not been studied yet, is the non-mandatory face masks use and the personality, psychological, dispositional, and political factors that can be involved. Only one study mentioned the use of face masks when not required by law^3^. The authors found that in the metropolitan city of Shanghai, 62% of the people involved in the investigation (N = 1282) were still using a mask in public spaces, even if no enforcement was present. It must be mentioned that in China the use of masks is quite common and usual, and could have become a behavioral norm, regardless of the pandemic^3^. Moreover, they suggested that face mask-wearing compliance is higher when mask use is enforced, but they did not analyze the personality, psychological, and dispositional factors leading to the choice of wearing a mask when non-required.

Most of the research on face mask use during the Covid-19 pandemic focused on the personality, psychological, dispositional, and political characteristics involved in the choice of using a mask, regardless of whether masks were required by Governments or not^1,4^. The majority of the studies refer to health organizations' preventative recommendations, not considering whether face mask use was enforced or not. Moreover, the following variables are among the most studied in the literature, as the main factors linked to the choice of wearing a mask during the Covid-19 pandemic.

According to psychological factors, fear of complications from Covid-19 disease, knowledge about the virus, and health consciousness are reported to be significantly and positively associated with the attitude toward wearing masks^5^. Mixed results are mentioned concerning the relationship between anxiety and depression, and adherence to precautionary measures during the pandemic, showing either a positive or negative association^6^. One study found that promoting reasoning is associated with greater face masks use^7^. Palmer et al. studied the embracing of masculine norms of toughness as a factor related to a negative approach toward the use of masks^4^. Moreover, empathy and subjective perception of mask normativity are related to a pro-mask position^1,8^.

Regarding dispositional issues studied by health psychology in the field of health-related attitudes, among others, reactance and trust in healthcare professions can be significant in the choice of wearing a mask or not^1^. High levels of reactance lead people to dislike being told how to behave and to do the opposite, as a matter of fact^9^. Previous evidence found that reactance is linked to less adherence to recommended health measures, and Mallinas et al. (2021) showed that reactance can be linked to the opposition to wearing a mask. Furthermore, the literature mentioned that trust in members of the scientific community is a factor related to adherence to scientific recommendations regarding the pandemic^10,11^. More specifically, trust in healthcare professions is reported to be positively associated with a pro-mask attitude^1^. In addition, resilience is becoming a key factor in the field of health psychology and has been studied for its psychological and physical outcomes during the Covid-19 pandemic, but, to our knowledge, not yet about the attitude toward mask-wearing^12–14^.

In relation to political factors, political orientation is widely reported in the literature on the attitude toward wearing a mask^15,16^ and other health-protective behaviors during the Covid-19 pandemic^17–19^ Indeed, using a face mask can become an ideological symbol, especially in those countries where the political parties’ opinion on the need to wear them or not is particularly divergent, as it was in the USA^20^. Some studies found that conservatism is associated with negative attitudes toward face mask use^1,10^. Capraro & Barcelo^7^ evidenced that right-leaning people seem to be more prone to wear masks when exposed to the message that Covid-19 is a threat to the community.

The present research focuses on the Italian situation. Recently, considerable literature has grown around the theme of the Covid-19 pandemic’s psychosocial impact in both nonclinical populations^21,22^ and special ones^23–27^. However, less is known about face mask use when enforced and non-enforced. The ISTAT annual report showed that Italy is among the most affected European countries, despite the great adherence levels to the health policies adopted by the government^28^. OMS data on January 2023 report a total of 185,417 deaths due to Covid-19 in Italy since the beginning of the pandemic^29^. Italy suffered severe consequences of the Covid-19 spread: several waves of the disease occurred, having the Government decided on strict lockdowns and safety rules that were periodically softened or suspended between 2020 and 202,130. The state of emergency was first declared on 31 January 2020 and then extended till the 31st of March 2022 (DL 24/2022). No strict lockdowns were lifted in 2022, which was a year of a gradual return to “normality”, also due to the vaccination campaign. When it comes to face mask use, starting from the 11^th^ of February 2022 they were no longer mandatory outdoors, except for crowded spaces, but still required in enclosed places. FFP2 masks had no longer been necessary from the 1^st^ of May even indoors, but they were still mandatory in public transport means, theatres, cinemas, concert halls, and healthcare settings. From 16 June FFP2 masks had to be worn only in public transport and healthcare facilities and services and from 30 September they are mandatory only in healthcare facilities^30^.

To our knowledge, no research has explored the psychological factors related to the choice of using a mask in Italy in periods of non-mandatory mask-wearing. Thus, the present study aimed to assess the psychological, political, and dispositional characteristics of people who chose to wear a mask and those who did not, in enclosed places, in 2022, when government rules no longer required it.

To start with, we compared two groups of people, those who were still using a mask and those who were not, in personality traits, psychological (anxiety and depression) and dispositional (trust in healthcare professions) factors, and Covid-19-related fears, controlling for demographic confounds (e.g. age and assigned sex at the birth)^31^. We have chosen to address both mental health facets, such as anxiety and depression, and cognitive perceptions, as trust in healthcare professions, concurrently, as we believe that comprehensively assessing these interconnected aspects provides a richer and more holistic understanding. Moreover, the choice of whether to wear the mask or not entails both the activation of cognitive and emotional processes: they are both involved in the formation of the final behavioral decision. We have chosen variables that are frequently discussed in the literature and are known to be closely associated with the decision to wear or not wear a mask. These variables were selected based on their perceived relevance to our study. As the literature claims that psychoticism is linked to less preventative health behaviors^32^, we expected to find higher levels of psychoticism in the first group. Based on the mentioned research, we also expected greater negative affectivity, antagonism, and psychoticism in the second group. Moreover, we anticipate higher anxiety, depression, trust in healthcare professions, and Covid-19-related fears for those who chose to wear face masks when non-mandatory^5,6^.

Secondly, we selected resilience, reactance, political orientation, and Covid-19 vaccinations as factors linked to the choice of wearing a mask or not^1,15,16^. We hypothesized that they may moderate the relationship between negative affectivity, as a personality trait, and the choice of wearing masks. Negative affectivity is reported to be correlated with depression, anxiety, and the tendency to react more sensitively to threat signals, like a pandemic^33^. Thus, it may affect the choice of wearing a mask. To our knowledge, no study tested similar models.

## Methods

### Participants

1151 adult participants were recruited (*M*_*age*_ = 32.4 years; *SD* = 12.1). Inclusion criteria were age 18 to 64 years and completion of the online survey. We collected data from 1268 participants; after applying inclusion criteria, 117 participants were excluded. Table 5 summarizes the sociodemographic characteristics of the final sample. Respectively 39% and 5.3% of the whole sample were still using a mask indoors and outdoors at the time of the research. The University of Padua Ethics Committee (Italy) on Psychological Research Areas (no. 4731/2022) approved the research study. Participants were fully informed and consented to the procedure, to which the committee agreed. This research was performed in accordance with the Declaration of Helsinki. All participants were informed that the data would be analyzed in an aggregated and anonymous way.

### Procedure

Participants were recruited through public announcements on social networks, local fliers, and word-of-mouth. The interview was implemented on the Qualtrics online platform, which could be accessed via a web link. The initial page of the online questionnaire showed an informed consent form, and the participant accessed the subsequent pages only after expressing the consent. The interview was made up of the following psychological tools: ad hoc Socio-Demographic Interview; Multidimensional Assessment of COVID-19-Related Fears (MAC-RF^34^); Personality Inventory for DSM-5 Personality Disorders-Brief Form (PID-5-BF^35^); Generalized Anxiety Disorder Scale-7 (GAD-7^36^); Patient Health Questionnaire-9 (PHQ-9^37^).

Data were collected from 30 June 2022 to 30 September 2022.

### Measures

#### Ad hoc socio-demographic interview

Assesses demographic characteristics (e.g., age, assigned sex at birth, education level, employment status), psychosocial attitudes (e.g., resilience, reactance, political orientation, trust in healthcare professions), and behavior associated with wearing masks (e.g., personal protection) from 30 June 2022 to 30 September 2022. More specifically, one item was used to assess resilience from the Brief Resilient Coping Scale by Sinclair^38^ (No matter what happens to me, I can control my reactions). Responses were on a 5-point Likert scale from 1 (It doesn’t describe me at all) to 5 (It describes me completely). The item assessing reactance had been already used by Dillard^39^ (The rules that require people to wear masks threaten my freedom of choice), and it was rated on a 5-point Likert scale from 1 (I strongly disagree) to 5 (I strongly agree). As to the political orientation, the item was previously used by Mahalik et al.^40^ (With which political orientation do you identify the most?), and it was rated on a 5-point Likert scale from 1, which indicated right-wing, to 5 which indicated left-wing political orientation. Furthermore, one item by Mallinas^1^ was used to assess trust in healthcare professions (I trust healthcare professions and their recommendations). The Likert scale ranged from 1 (I strongly disagree) to 5 (I strongly agree). Moreover, participants were asked if they were still using a facemask in closed environments despite it being no more required by the Government's rules. They answered 1 for yes and 2 for no. Lastly, the survey included an item to assess if people had had a Covid-19 vaccination or not (Did you get a Covid-19 vaccination?).

#### Assessment of COVID-19-related fears (MAC-RF^34^)

It is an 8-item self-report tool designed to assess clinically relevant fear related to COVID-19, related to the past week, derived from The items refer to fear to (for) the body and others, of (not) knowing, and of (in)action (e.g., *“I don’t trust my own body to protect me against the coronavirus infection”*). Each item is evaluated through a five-point Likert response scale (1 = Very unlike me to 5 = Very like me). The total score on the test can vary from 0 to 32, where higher scores indicate greater fears connected to Covid. The tool showed good internal consistency (*Cronbach’s alpha* = 0.84) and satisfactory split-half reliability (*Spearman-Brown r* = 0.78). In the present study, the Cronbach’s alpha was α = 0.80.

#### Personality inventory for DSM-5 personality disorders-brief form (PID-5-BF^35^)

It is a 25-item self-report tool evaluating the five pathological personality traits, namely, negative affectivity, detachment, antagonism, disinhibition and psychoticism (e.g., “*People would describe me as reckless*”). Each item is evaluated through a four-point Likert response scale (0 = never to 3 = always). The total score on the test can vary from 0 to 75, where the higher the score the greater the dysfunction in the specific personality trait domain. The tool showed good internal consistency. In the present study, the Cronbach’s alphas for negative affectivity, detachment, antagonism, disinhibition, and psychoticism were respectively α = 0.59, α = 0.62, α = 0.62, α = 0.71, α = 0.69.

#### Generalized anxiety disorder scale—7 (GAD-7^36^)

It is a 7-item self-report tool evaluating worry and anxiety symptoms experienced in the last 2 weeks (e.g., “*Worrying too much about different things*”). Each item is evaluated through a four-point Likert response scale (0 = not at all 3 = nearly every day). The total score on the test can vary from 0 to 21, where the higher the score the greater the severity of anxiety. The tool showed excellent internal consistency (*Cronbach’s alpha* = 0.92) and test–retest good reliability (*intraclass r* = 0.83). In the present study, the Cronbach’s alpha was α = 0.90.

#### Patient health questionnaire—9 (PHQ-9^37^)

It is a 9-item self-report tool evaluating severity of depressive symptoms experienced in the last 2 weeks (e.g., “*Little interest or pleasure in doing things*”). Each item is evaluated through a four-point Likert response scale (0 = not at all to 3 = nearly every day). The total score on the test can vary from 0 to 27, where the higher the score the greater the severity of depression. The tool showed an excellent internal consistency (*Cronbach’s alpha* = 0.89). In the present study, the Cronbach’s alpha was α = 0.87.

### Data analysis

Data were analyzed using the statistical software jamovi 2.0 and IBM SPSS Statistics 29.0.1.0 (Retrieved from https://www.jamovi.org and https://www.ibm.com/products/spss-statistics). First, we reported statistical descriptives.

To identify potential differences in five-dimensional personality traits (PID-5-BF), anxiety and depression (GAD-7, PHQ-9), trust in healthcare professions, and COVID-related fears (MAC-RF) in groups wearing vs not wearing a mask, we ran a Multivariate Analysis of Covariance (MANCOVA) models with negative affectivity, antagonism, psychoticism, depression, anxiety, trust in healthcare professions, and fears as our DVs, controlling for the effect of the age and assigned sex at birth.

To gain a deeper understanding of the potential influence of psychological factors (related to resilience, reactance, and political orientation) on wearing a mask (DV), we performed three models of moderation analysis with negative affect (personality trait; IV) as a predictor and psychological characteristics as moderators.

Finally, we perform an additional moderation analysis to test the modulatory effect of Covid-19 vaccination between negative affectivity (predictor) and wearing a mask (DV).

## Results

### One-way multivariate analysis of covariance (MANCOVA)

A one-way MANCOVA was run to test whether dependent variables (DVs) of personality traits (namely negative affectivity, antagonism, psychoticism; PID-5-BF total scores), psychological and dispositional factors (anxiety GAD-7 total score, depression PHQ-9 total score, trust in healthcare professions), and COVID-related fears (MAC-RF total score), differ significantly in the individual wearing and not wearing the mask (IV), after controlling for age and assigned sex at birth. The independent variable was wearing a mask (2-level: Yes, No). S tatistically significant result s were found between the DVs and the I V (fixed factor) after controlling for age and assigned sex at birth(F(7, 1125) = 18.888, *p* < 0.00 0, Wilks' Λ = 0.895, partial η2 = 0.105). To test the impact of the effect on the individual DVs, a univariate F-test using an alpha level of 0.05 was performed. The main effect of wearing a mask was significant on negative affectivity (F(1, 1131) = 11.167, *p* < 0.001, partial η2 = 0.010), psychoticism (F(1, 1131) = 5.487, *p* = 0.019, partial η2 = 0.005), anxiety (F(1, 1131) = 7.144, p = 0.008, partial η2 = 0.006), trust in healthcare professions (F(1, 1131) = 39.039, *p* < 0.000, partial η2 = 0.033) and COVID-related fears (F(1, 1131) = 83.660, *p* < 0.000, partial η2 = 0.069). Overall, the Mancova analysis showed small to medium effect sizes (below the threshold of η2 < 0.06) of the independent variable on the dependent variables. Pair-wise comparison followed by a univariate F-test indicates that independently by age and assigned sex at birth, individuals wearing the mask were characterized by a higher level of negative affectivity (*M*_Mask(YES)_ = 6.050 ± 0.132, *M*_Mask(NO)_ = 5.478 ± 0.106, *p* < 0.001, C.I. [0.236 0.909]), a higher level of psychoticism (*M*_Mask(YES)_ = 4.56 7 ± 0.137, *M*_Mask(NO)_ = 4.149 ± 0.111, *p* = 0.019 , C.I. [0.068 0.769]), a higher level of anxiety (*M*_Mask(YES)_ = 7.690 ± 0.224, *M*_Mask(NO)_ = 6.913 ± 0.180, *p* = 0.008, C.I. [0.207 1.3 48]), a higher level of trust in healthcare professions (*M*_Mask(YES)_ = 4.683 ± 0.035, *M*_Mask(NO)_ = 4.402 ± 0. 028, *p* < 0.000, C.I. [0.193 0.369]) and a higher level of COVID-related fears (*M*_Mask(YES)_ = 12.52 8 ± 0.280, *M*_Mask(NO)_ = 9.194 ± 0.226, *p* < 0.000, C.I. [2.619 4.049]).

### Moderation analyses

A moderation test was run, with negative affectivity (model 1) as the predictor, wearing a mask indoors as the dependent variable, and resilience (Item: *No matter what happens to me, I can control my reactions*) as a moderator.

**Figure 1:**
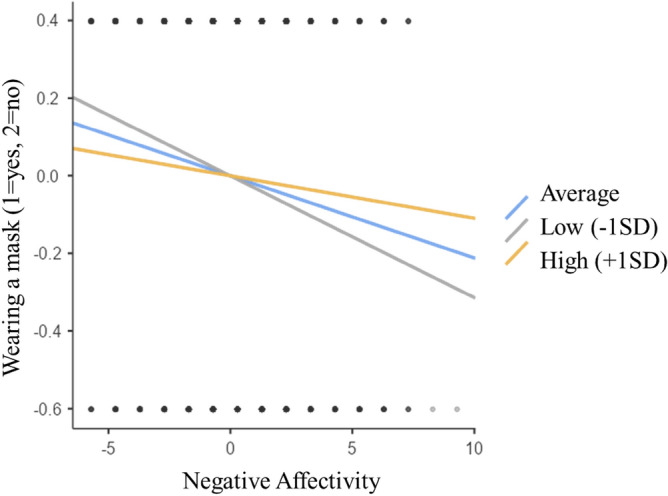
Slop plots of the effect of the predictor negative affectivity on the dependent variable wearing (no, yes) a mask at different levels of the moderator resilience.

*Model 1* There was a significant main effect between negative affectivity and wearing a mask, *b* = − 0.21, CI [− 0.03, − 0.01], z = *−* *4.18*, *p* < 0.001, and a significant main effect of resilience on wearing a mask,* b* = − 0.05, CI [− 0.08, − 0.01], z = − 2.52, *p* = 0.012. A significant interaction was also found by resilience on negative affectivity and wearing a mask, *b* = 0.01, CI [0.00, 0.06], z = 2.13, *p* = 0.033. Participants who reported lower than average levels of resilience experienced a greater effect of negative affectivity on not wearing a mask (*b* = -0.03, CI [− 0.04, − 0.08], z = − 4.60, *p* < 0.001), when compared to the average (*b* = − 0.02, CI [− 0.03, − 0.01], z = − 4.18, *p* < 0.001). No significant effect on participants who reported higher levels of resilience. Results suggested that the effect of negative affectivity on wearing a mask is partially moderated by resilience (Table 1; Fig. 1).

**Table 1:** Simple slop estimates of the predictor (negative affectivity) on the dependent variable (wearing a mask: yes, no) at different levels of the moderator (resilience).

	Estimate	SE	95% confidence interval		*Z*	*P*
			Lower	Upper		
Average	− 0.0211	0.00506	− 0.0311	− 0.0112	− 4.18	< .001
Low (− 1SD)	− 0.0314	0.00682	− 0.0447	− 0.018	− 4.60	< .001
High (+ 1SD)	− 0.0109	0.00712	− 0.0249	0.00305	− 1.53	0.126

Three additional moderation tests were run, with negative affectivity as the predictor and wearing a mask (indoors) as the dependent variable, and reactance (*The rules that require people to wear masks threaten my freedom of choice*) (model 2), political orientation (right-to-left spectrum; Likert Scale range from 1 = right to 5 = left) (model 3) and COVID-19 vaccination (yes = 1, no = 2) (model 4) as moderators.

*Model 2* There was a significant main effect between negative affectivity and wearing a mask, *b* = − 0.02, CI [− 0.03, − 0.01], z = *−* *4.04*, *p* < 0.001, and a significant main effect of reactance on wearing a mask* b* = 0.11, CI [0.09, 0.13], z = 9.88, *p* < 0.001. A nonsignificant interaction was also found by reactance on negative affectivity and wearing a mask, *b* = 0.004, CI [− 0.00, 0.01], z = 1.01, *p* = 0.311. Results suggested that the effect of negative affectivity on wearing a mask is not moderated by reactance (Table 2).

**Table 2:** Simple slop estimates of the predictor (negative affectivity) on the dependent variable (wearing a mask: yes, no) at different levels of the moderator (reactance).

	Estimate	SE	95% confidence interval		*Z*	*p*
			Lower	Upper		
Average	− 0.0196	0.00487	− 0.0292	− 0.01009	− 4.03	< .001
Low (− 1SD)	− 0.0243	0.00677	− 0.0376	− 0.01105	− 3.59	< .001
High (+ 1SD)	− 0.0149	0.00667	− 0.0280	− 0.00186	− 2.24	0.025

*Model 3* There was a significant main effect between negative affectivity and wearing a mask, *b* = − 0.02, CI [− 0.03, − 0.01], z = *−* *3.03*, *p* = 0.002, and a significant main effect of political orientation on wearing a mask* b* = − 0.07, CI [− 0.10, − 0.03], z = − 4.11, *p* < 0.001. A nonsignificant interaction was also found by political orientation on negative affectivity and wearing a mask, *b* = 0.004, CI [− 0.01, 0.02], z = 0.83, *p* = 0.406. Results suggested that the effect of negative affectivity on wearing a mask is not moderated by political orientation (Table 3).

**Table 3:** Simple slop estimates of the predictor (negative affectivity) on the dependent variable (wearing a mask: yes, no) at different levels of the moderator (political orientation).

	Estimate	SE	95% confidence interval		*Z*	*p*
			Lower	Upper		
Average	− 0.0155	0.00511	− 0.0255	− 0.00548	− 3.03	0.002
Low (− 1SD)	− 0.0197	0.00695	− 0.0333	− 0.00610	− 2.84	0.005
High (+ 1SD)	− 0.0113	0.00744	− 0.0259	0.00329	− 1.52	0.129

*Model 4* There was a significant main effect between negative affectivity and wearing a mask, *b* = − 0.02, CI [− 0.03, − 0.01], z = *−* *3.43*, *p* < 0.001, and a significant main effect of having COVID-19 vaccination on wearing a mask* b* = 0.24, CI [0.06, 0.42], z = 2.66, *p* = 0.008. A nonsignificant interaction was also found by COVID-19 vaccination on negative affectivity and wearing a mask, *b* = 0.02, CI [− 0.04, 0.08], z = 0.63, *p* = 0.530. Results suggested that the effect of negative affectivity on wearing a mask is not moderated by having COVID-19 vaccination (Table 4).

**Table 4:** Simple slop estimates of the predictor (negative affectivity) on the dependent variable (wearing a mask: yes, no) at different levels of the moderator (COVID vaccination).

	Estimate	SE	95% confidence interval		*Z*	*p*
			Lower	Upper		
Average	− 0.0173	0.00504	− 0.0272	− 0.00740	− 3.43	< .001
Low (− 1SD)	− 0.0205	0.00716	− 0.0345	− 0.00645	− 2.86	0.004
High (+ 1SD)	− 0.0141	0.00718	− 0.0282	− 9.29e−6	− 1.96	0.050

## Discussion

The medical, psychological, and socio-political implications of the COVID-19 pandemic have been discussed in depth in the literature. To prevent the spread of the virus, the Italian government instituted a series of decrees where, among others, PPE was normed, during the two-year pandemic. However, even when the mandatory restrictions were removed, some people felt comfortable maintaining certain behaviors; thus, social distancing and mask-wearing emerged as new behavioral norms^41^. The present research aimed to understand the psychological, political, and dispositional characteristics of Italian people who chose to wear a mask and those who did not when government rules no longer required it.

As a preliminary step, we found that despite the norm’s suspension, of the 1151 people in the present sample 39% still wore masks indoors and a smaller portion 5.3% even outdoors.

Regarding our first hypothesis, as expected, the present results showed that people who wear a mask indoors after the end of government-imposed rules showed higher levels of psychoticism and negative affectivity. Literature defines psychoticism personality traits as associated with less adaptive behaviors and fixed response patterns. People high in psychoticism may be more in trouble with changing the behavior of wearing a mask, which can have become a reassuring habit. The result can suggest how hard they struggle to adapt to changing situations^32^. Also, negative affectivity was greater in participants who were still using a mask indoors when non-mandatory in the present study. People with this personality characteristic show more concerns and worries about their health and are more likely to maintain good health habits, like wearing a mask to protect themselves from contagion^42,43^. Considering the other personality traits, no significant differences emerged. Antagonism seems not to be related to the choice of wearing a mask: it is characterized by low honesty-humility, and low emotionality, aspects that may not be primarily involved in the mentioned choice^44^. Thus, people with high psychoticism and negative affectivity may have difficulty and lower adaptability to go beyond the norm and return to a baseline condition. Moreover, higher anxiety was found in those who were still wearing a mask when non-required. Depression was not confirmed by controlling for age and assigned sex at birth. Mixed results are reported in the literature when it comes to the association between anxiety and depression and adherence to the precautionary rules during the pandemic^6^. Our results align with the literature claiming that anxiety may be significantly linked to the behavior of wearing masks. People with high levels of anxiety may find it hard to quit the behavior of wearing masks, as having internalizing symptoms frequently leads to reacting more sensitively to stressful events and threat signals, as a pandemic^33,45^. A possible further reading could be related to the fact that people who deviate from the new norm, such as wearing masks when others do not, may experience greater anxiety about the potential risk of triggering discrimination. It is important to point out that individual factors turn out to have small effects as opposed to contextual effects which have medium effects. Moreover, factors not assessed in the present study, such as perceived risk due to health status and potential coronavirus exposure, may better account for the majority of the variance in coronavirus-related anxiety^46^. Also, trust in healthcare professions seems to be a factor related to the choice of wearing masks: people who were still wearing a mask showed to rely more on healthcare professions’ recommendations^1^.

When it comes to the COVID-19-related fears, results are in line with our hypothesis: people who were still wearing masks indoors had greater worries about the pandemic. Fear of complications from Covid-19 disease is reported to be significantly and positively associated with the attitude toward wearing masks^5^.

Considering our second hypothesis, the findings only partially met the expected results. In line with expectations, high levels of negative affectivity were associated with the choice of wearing a mask indoors. Participants who reported lower-than-average levels of resilience experienced a more significant effect of negative affectivity on the behavior of not wearing a mask indoors*,* whereas higher-than-average levels did not differentiate from average levels of resilience. As a qualitative indication of resilience, we considered the personal belief that, independently of what happens, individuals feel able to control their own reactions. A good ability to control one's reactions to environmental changes, especially exposure to stress, is an intrinsically emotional experience. An individual's ability to regulate their reactions is essential to their susceptibility or resilience to adversity^47^. Resilience is a complex construct, and many individual, social, cognitive, emotional, and behavioral factors come into play and interact with each other^48^. One of the most consistent findings in stress and resilience research is that the more controllable a stressful situation is the better people cope with it^49,50^. A sense of resilience given by the ability to control one's reactions in different situations after stressful situations cannot disengage from the personality trait linked to negative affectivity, which together can shape individual behavior in post-stressful situations such as the current post-pandemic scenario that we are all still facing. However, we have not investigated the personal meaning of wearing a mask, so our suggestions must be considered cautiously.

In contrast, behavioral control mechanisms, such as reactance, political orientation, and experience of having a COVID-19 vaccination, did not significantly moderate the relationship between negative affectivity and the choice to wear or not wear a face mask indoors. One possible interpretation is that behavior control mechanisms, as opposed to maladaptive personality traits, influence mask-wearing behavior in differentiated social contexts and/or in the presence of external rules that limit personal choices^51,52^. Along this line, psychological attitudes such as reactance and political orientation might more likely influence individual behaviors when more oriented towards some otherness^53^, thus such social attitudes might play a marginal role in an intrinsically personal choice to spontaneously wear a face mask during the post-pandemic period of COVID-19. Finally, receiving or not receiving a COVID-19 vaccination did not moderate the effect of negative affectivity on wearing a mask indoors. We cannot clarify the lack of effect from our findings because we did not differentiate the personal meanings of mask-wearing. Further research should consider the subjective significance of wearing a face mask indoors and outdoors to better elucidate the potential interaction between individual personality traits and psychosocial attitudes in continued indoor facemask use and personal safety.

To our knowledge, the present study is the first to assess the personality, psychological, dispositional, and political factors involved in using a mask in Italy in periods of non-mandatory mask-wearing. Despite the contribution that our study brings to the understanding of post-pandemic behavioral processes, our work has some limitations. First, the present study has a cross-sectional design at a one-time point; a longitudinal perspective might allow us to test the hypotheses' stability or movement over time. A second point to stress among the limitations is our convenient sample that may only represent some Italian regions while others are underrepresented (see Table 5 for detailed sociodemographic characteristics). Similarly, most of the participants had medium to high education levels. Future studies should overcome these limitations by collecting data on a larger and more representative sample. Future research may also focus on other factors, not considered in the present study, that may influence the choice of wearing a mask or not when non-mandatory, for example, have lost someone due to the pandemic, or have a chronic disease. An additional limitation to note is that the study was conducted exclusively through online surveys. It is likely that a mixed mode of online survey and paper–pencil method could enable it to reach people who are unfamiliar with the use of devices or do not have online access or access to their e-mail/phone. Even if the interviewer's influence, which does not exist in the online administration, ensured anonymity, where questions of a sensitive nature were answered more readily, associating the use of the paper–pencil could allow the support of an operator available to answer any questions that may arise during the process.

**Table 5:** Sociodemographic characteristics of participants. * 16 participants (1.4%) did not respond to the ethnicity item.

Variable	*n*	*%*	Variable	*n*	*%*
Assigned sex at birth			Ethnicity*		
Female	768	66.7	Caucasian	1101	97.1
Male	382	33.2	Asian	5	0.5
Other	1	0.1	Arabic	1	0.1
			Black latin	1	0.1
Gender identity			Other	27	2.7
Cisgender	1089	94.6			
Transgender persons	4	0.3	Educational level		
Non-binary persons	26	2.3	Lower secondary	59	5.1
Other	32	2.8	Upper secondary	468	40.7
			Bachelor's	307	26.7
Sexual orientation			Master's	304	26.4
Gay/lesbian	41	3.6	Doctorate	12	1
Bisexual	43	3.7	Other	1	0.1
Pansexual	12	1.0			
Asexual	11	1.0	Work status		
Heterosexual	1038	90.2	Student	281	24.4
Other	6	0.5	Student-worker	28	2.4
			Worker	754	65.5
Region of residence			Retired	16	1.4
Centre	75	6.5	Unemployed	56	4.9
North-West	290	25.1	Other	16	1.4
North-East	699	60.8			
South	65	5.6	COVID vaccination		
Islands	9	0.8	Yes	1121	97.4
Other	13	1.1	No	30	2.6

It should be noted that a group of 5.3% of participants used masks outdoors. However, the study aimed to investigate how individuals who still used the mask and those who did not use it differed in personality traits, psychological and dispositional factors. Our sample size did not allow for further demographic or psychological stratification into groups based on the combination of factors (for example, wearing or not wearing a mask indoors and outdoors). Subsequent studies would first need to replicate the current findings that refer to general factors that potentially shape the behavior of all people and then consider greater population stratification.

The findings of this study provide a better understanding of individuals’ responses to post-pandemic changes. The COVID-19 pandemic has had countless implications on health and social settings; an element that should not be overlooked concerns the personal and contextual features that can make the process of returning to everyday life harder for some people. Studying these aspects may help to develop support programs and to provide adequate care for people who need it the most, also in post-pandemic times. Moreover, it may help the scientific community to understand the characteristics of people who tend not to follow healthcare guidelines and develop preventative systems.

## Data Availability

The data that support the findings of this study are available from the corresponding author, [SS], upon reasonable request.
